# Paradoxical gender effects in meat consumption across cultures

**DOI:** 10.1038/s41598-024-62511-3

**Published:** 2024-06-13

**Authors:** Christopher J. Hopwood, Jahn N. Zizer, Adam T. Nissen, Courtney Dillard, Andie M. Thompkins, Joāo Graça, Daniela Romero Waldhorn, Wiebke Bleidorn

**Affiliations:** 1 University of Zurich, Zurich, Switzerland; 2 University of California, Davis, Davis, USA; 3 Mercy For Animals, Los Angeles, USA; 4 University of Groningen, Groningen, The Netherlands; 5 Rethink Priorities, Barcelona, Spain

**Keywords:** Paradoxical gender effect, Gender, Meat consumption, Equality, Culture, Human behaviour, Environmental social sciences

## Abstract

Men tend to eat more meat than women, but it is not clear why. We tested three hypotheses in a cross-cultural design (20,802 individuals in 23 countries across four continents): that gender differences are (a) universal, (b) related to gender roles and thus weaker in countries with higher gender equality and human development, or (c) related to opportunities to express gender roles and thus stronger in countries with higher gender equality and human development. Across all countries, men tended to consume more meat than women. However, this difference increased significantly in countries with greater human development and gender equality. The paradoxical gender gap in meat consumption aligns with previous research that suggests greater differences in behavior across genders in contexts that are more developed and gender equal. We discuss implications for theories of culture and gender as well as practical implications for global meat reduction.

## Introduction

Meat consumption represents a significant threat to environmental sustainability via its impact on climate change, human health via its association with risk for disease, and social justice via its impact on animal welfare worldwide^1–5^. Meat consumption is reliably higher among men than women in North American and European samples^6–9^, but reasons for this difference remain unclear. Understanding this difference can shed light on how gender interacts with culture and may pave ways to reduce meat consumption and its negative impacts. We used data from 23 countries from four continents to test three competing hypotheses about gender differences in meat consumption.

The first hypothesis is that gender differences in meat consumption frequency are universal. Universalism is typically tied to arguments related to biological sex differences in dietary needs that operate independent of culture and context^10^. For instance, Fessler^11^ argued that women experience hormonally-mediated immunosuppression during menstruation and pregnancy that leads them to avoid meat consumption. Other theorists have speculated about differential evolutionary pressures^12^. For instance, it has been hypothesized that mean prefer meat because they are more likely to behave in a way that risks injury^13^, and the profile of amino acids provided by animal flesh promotes muscle and bone strength that protects against injury^14^. Similarly, evolved social norms in which men tend to be rewarded for being good hunters^15^ could have contributed to its being more valued, both in terms of taste and social status, among men.

Indeed, cultural and economic dynamics have played an important role in meat consumption throughout history^16^. In many cultures, meat consumption is linked to masculinity and masculine identity^9,17–19^. There is evidence that vegetarian men are viewed as less attractive than omnivorous men in some cultures^20^, demonstrating that cultural differences connect gender norms with masculinity-linked behaviors. Thus, a second hypothesis is that meat consumption frequency is influenced by cultural norms that associate meat consumption with positive traits, such as masculinity and virility for men, and thus would be higher in countries with stronger gender role norms.

The third hypothesis is less intuitive. Previous research has identified paradoxical gender effects, such that gender differences in certain psychological variables such as personality traits^21,22^, interests^23^, and self-esteem^24^ are actually greater in countries with greater gender equality. Two possible explanations have been offered for this paradoxical gender effect. First, it has been suggested that gender equality, being associated with greater wealth and increased purchasing power^25–27^, provides opportunities for natural expression of certain behaviors^22,28^. In this case, gender differences in meat consumption frequency would be greater in countries with greater gender equality, to the extent that there are universal differences in meat consumption preferences across genders.

A second possible explanation involves reference group effects^29^. In countries with higher gender equality, people responding to questions about themselves may be more likely to compare themselves to the general population, whereas in countries with lower gender equality, respondents may be more likely to compare themselves to others of their same gender^30^. This would have the effect of obscuring actual gender differences in less gender-equal countries. Given that the frequency of meat consumption is a more behavioral criterion than those for which paradoxical gender effects have been observed, such as self-reported personality traits, interests, or self-esteem, comparisons of meat consumption rates across cultures should be less prone to reference group effects.

Understanding cross-cultural differences in the meat consumption gender gap may provide insights regarding the role of culture in gender differences and test the limits of paradoxical gender effects that have been observed for other psychological variables. It may also provide clues about how to reduce meat consumption globally. If gender differences in meat consumption are universal, strategies designed to reduce meat consumption may vary across genders^31^, but the gender-specificity of such strategies would not depend on culture. Finding that gender differences in meat consumption were stronger in countries with lower gender equality would highlight the role of masculinity and related stereotypes in reducing meat consumption among men^20^. A paradoxical gender effect in which gender differences in meat consumption are higher in more developed countries would suggest that meat consumption is a function of social opportunity and highlight the potential effectiveness of different kinds of strategies in different cultural contexts. We note that gender equality and human development are different in many ways, but they are related empirically and intertwined in terms of our hypotheses. Gender equality is generally afforded by aspects of human development, so to the extent that gender differences in meat consumption are moderated by one of these factors they would likely be moderated by both. However, the use of both indexes allows us to begin to examine the specificity of gender equality relative to human development more generally. At the same time, it must be acknowledged that with the modest number of countries included in this study, a test of the differences in effects between these variables is quite preliminary.

In summary, the purpose of this study was to use cross-cultural data to examine gender differences in meat consumption frequency. We expected men to consume more meat than women, on average, across all countries, and people in more developed and gender-equal countries to eat more meat. Our primary focus was on testing whether gender differences across countries would (a) not differ across countries, (b) be weaker in countries with higher gender equality and human development, or (c) be stronger in countries with higher gender equality and human development.

## Method

### Sample

All methods were performed in accordance with the relevant ethical guidelines and regulations. Participants from 23 countries on four continents (see Table 1 for list of included countries) were recruited in 2021 using a stratified sampling approach created by the survey platform Cint. We recruited 1000 participants per country such that participants from each country were representative of the nation in terms of gender and age. Among the 28,229 participants who provided any data, the research team excluded participants who did not correctly respond to validity checks (e.g., not selecting the option ‘apple’ after being instructed to do so), provided nonsense responses to open-ended questions, or did not complete the survey (n = 7263). We then reopened the survey to more potential participants in an effort to get as close as possible to our target of 1000 participants per country, although our success was variable (see country-level sample sizes in Table 1). We also removed participants who did not identify as a man or woman (n = 164) for this study. This resulted in a sample of 20,802 participants. For more information about the CINT survey platform, the measures included in this study, and other unrelated tasks that were completed by the participants, see https://osf.io/tx3u9/?view_only=07504017687249d3bb05d93db9c83914.

**Table 1:** Sample countries with predictor scores and gender effects.

Country	N	%M	Country level predictor		Meat consumption score (land animals)			Cohen’s *d* (F < M) [CI 95%]
			HDI	GGGI	M	F	Total	
Argentina	942	50.00	0.842	0.752	4.47	3.62	4.05	0.49 [0.36, 0.62]
Brazil	983	49.14	0.754	0.695	4.59	4.03	4.30	0.30 [0.17, 0.42]
Canada	867	47.06	0.936	0.772	4.46	3.80	4.11	0.36 [0.23, 0.49]
Chile	932	49.25	0.855	0.716	4.16	3.51	3.83	0.37 [0.24, 0.50]
China	884	49.43	0.768	0.682	4.61	4.60	4.61	− 0.01 [− 0.14, 0.12]
Colombia	975	47.79	0.752	0.725	4.84	4.21	4.51	0.38 [0.25, 0.50]
France	909	46.97	0.903	0.784	4.60	4.04	4.30	0.35 [0.22, 0.48]
Germany	968	47.62	0.942	0.796	3.76	2.84	3.28	0.58 [0.45, 0.71]
India	894	55.82	0.633	0.625	1.85	1.92	1.88	− 0.03 [− 0.16, 0.10]
Indonesia	987	50.96	0.705	0.688	3.30	3.24	3.27	0.04 [− 0.08, 0.17]
Italy	947	49.00	0.895	0.721	4.09	3.65	3.86	0.28 [0.15, 0.40]
Japan	553	49.55	0.925	0.656	4.49	4.21	4.35	0.18 [0.02, 0.35]
Korea	917	50.05	0.925	0.687	4.03	3.71	3.87	0.21 [0.08, 0.34]
Malaysia	932	51.72	0.803	0.676	3.86	3.58	3.72	0.17 [0.04, 0.30]
Mexico	973	48.61	0.758	0.757	4.43	3.91	4.16	0.32 [0.19, 0.45]
Netherlands	903	47.95	0.941	0.762	4.47	4.00	4.23	0.28 [0.15, 0.41]
Poland	962	47.71	0.876	0.713	4.36	3.67	4.00	0.45 [0.32, 0.58]
Russia	959	46.51	0.822	0.708	4.78	4.16	4.45	0.35 [0.22, 0.48]
Singapore	730	46.85	0.939	0.727	4.62	3.97	4.28	0.36 [0.22, 0.51]
Spain	971	50.88	0.905	0.788	4.71	4.37	4.54	0.21 [0.09, 0.34]
Thailand	942	48.94	0.800	0.710	5.19	4.66	4.92	0.26 [0.13, 0.39]
UK	828	50.85	0.929	0.775	4.14	3.35	3.75	0.42 [0.28, 0.56]
USA	862	48.61	0.921	0.763	4.76	4.35	4.55	0.21 [0.08, 0.35]

Effect sizes for the whole sample including all individuals (N = 20,802). HDI = Human Development Index. GGGI = Global Gender Gap Index.

### Measures

Respondents rated the frequency of their consumption of various classes of food from "*1* = *Never"* to "*11* = *Two or more times per day".* We calculated land animal consumption by averaging scores for the categories "Cow/beef", "Pig/Pork", "Chicken or other fowl", and "Other land animals". As the items measure frequency within a category rather than portion size, we assumed that they represent the amount of meat eaten without being greatly influenced by gender differences in general caloric intake.

We used the Human Development Index (HDI), a multi-scale measure of human development that is published by the United Nations^32^, to rank countries according to their level of development on three dimensions: health (as measured by life expectancy at birth), education (as measured by the years of schooling and the expected years of schooling), and standard of living (as measured by gross national income per capita). This index is viewed as superior to the Global Domestic Product (GDP) as a measure of development as it, unlike the latter, represents not only average growth in income but also considers health and education as the key components of human development^33^. The data were derived manually from the website of the United Nations Development Program in January of 2023, where the data from 2021 were freely available.

We used the Global Gender Gap Index (GGGI), a multi-scale measure of gender equality that is published by the World Economic Forum^34^, to assess national differences in gender equality. The score is calculated based on four indicators: economic participation and opportunity, educational attainment, health and survival, and political empowerment. The data from 2021 were derived from the Global Gender Gap Report 2021, which was published by the World Economic Forum in 2021.

### Analyses

We first examined mean levels of study variables across countries to provide an initial indication of overall levels of meat consumption frequency and gender differences across countries. We then tested hypotheses using multi-level intercept-and-slopes-as-outcome models with level 1 individual- and level 2 country-level predictors. We constructed increasingly complex models, starting with an intercept-only model and ending with a cross-level interaction model. All our models were constructed to be nested within less complex ones, giving us the ability to statistically compare the variance explained by the more complex models. In the first step, an intercept-only model was constructed to investigate the ratio of the variance of meat consumption frequency that the country levels alone explain and to test whether our multi-level approach was necessary and justified. This model was specified as:

1 $${\text{Y}}_{{{\text{ij}}}} = {\upgamma }_{00} +\upmu _{{0{\text{j}}}} + {\text{r}}_{{{\text{ij}}}}$$

with Y_ij_ representing the combined meat consumption score for each individual* i* in each nation *j*, which is composed of γ_00_, the average standardized meat consumption score across the population of all countries *j* (i.e., the overall mean), μ_0j_, the individual deviation of each country *j* form this mean, and each Individual *i*'s deviation from these means, r_ij_.

In the next step, we added the Level 1 predictors of binary gender, linear age, and quadratic age, to compose a random intercept model predicting meat consumption. We included age because of research indicating that meat consumption varies across age, peaking at age 20–49 with lower consumption at younger and older ages^8^. The random intercept model was specified as:

2 $${\text{Y}}_{{{\text{ij}}}} =\upgamma _{00} +\upgamma _{{{1}0}} \left( {{\text{gender}}_{{{\text{ij}}}} } \right) +\upgamma _{{{2}0}} \left( {{\text{age}}_{{{\text{ij}}}} } \right) +\upgamma _{{{3}0}} \left( {{\text{age}}_{{{\text{ij}}}}^{{2}} } \right) +\upmu _{{0{\text{j}}}} + {\text{r}}_{{{\text{ij}}}}$$

with γ_10_ being added to the equation as the regression slopes for gender across countries, and γ_20_ and γ_30_ as the regression slopes for age and quadratic age across countries. In this model, intercepts can vary randomly, with slopes remaining constant across countries.

Next, we tested a random coefficients model, for which we added the random coefficients for the slopes of gender, age, and the quadratic term of age. As described below, the age slopes did not explain additional variance. The resulting random coefficients model was specified as:

3 $${\text{Y}}_{{{\text{ij}}}} =\upgamma _{00} +\upgamma _{{{1}0}} \left( {{\text{gender}}_{{{\text{ij}}}} } \right) +\upgamma _{{{2}0}} \left( {{\text{age}}_{{{\text{ij}}}} } \right) +\upgamma _{{{3}0}} \left( {{\text{age}}_{{{\text{ij}}}}^{{2}} } \right) +\upmu _{{0{\text{j}}}} +\upmu _{{{\text{1j}}}} \left( {{\text{gender}}_{{{\text{ij}}}} } \right) + {\text{r}}_{{{\text{ij}}}}$$

with μ_1j_ added as the varying slope of gender in each country *j*, representing how the extent of gender effects varies across countries.

In a final step, we added the human development and gender equality indices as moderators of the gender effect into the equation to create an intercepts-and-slopes-as-outcome model with a cross-level-interaction. These variables correlated *r*. = 63 in our data. We added them as variables in separate models to avoid multicollinearity.

The final models were specified as:

4 $$\begin{aligned} {\text{Y}}_{{{\text{ij}}}} & =\upgamma _{00} +\upgamma _{{{1}0}} \left( {{\text{gender}}_{{{\text{ij}}}} } \right) +\upgamma _{{{2}0}} \left( {{\text{age}}_{{{\text{ij}}}} } \right) +\upgamma _{{{3}0}} \left( {{\text{age}}_{{{\text{ij}}}}^{{2}} } \right) +\upgamma _{{0{1}}} \left( {\text{moderator j}} \right) \\ & \quad +\upgamma _{{{11}}} \left( {\text{moderator j}} \right) \times \left( {{\text{gender}}_{{{\text{ij}}}} } \right) +\upmu _{{0{\text{j}}}} +\upmu _{{{\text{1j}}}} \left( {{\text{gender}}_{{{\text{ij}}}} } \right) + {\text{r}}_{{{\text{ij}}}} \\ \end{aligned}$$

with moderator representing HDI for the model containing human development, GGGI for the model containing gender equality, γ_01_ representing the effects of development/equality across countries, and γ_11_ for the effect of the cross-level-interaction of development/equality and gender across countries and individuals.

### Ethics

This study was deemed exempt from formal review according to the criteria of the University of Zurich Faculty of Arts and Sciences Ethics Commission. All participants provided informed consent to participate.

## Results

**Figure 1:**
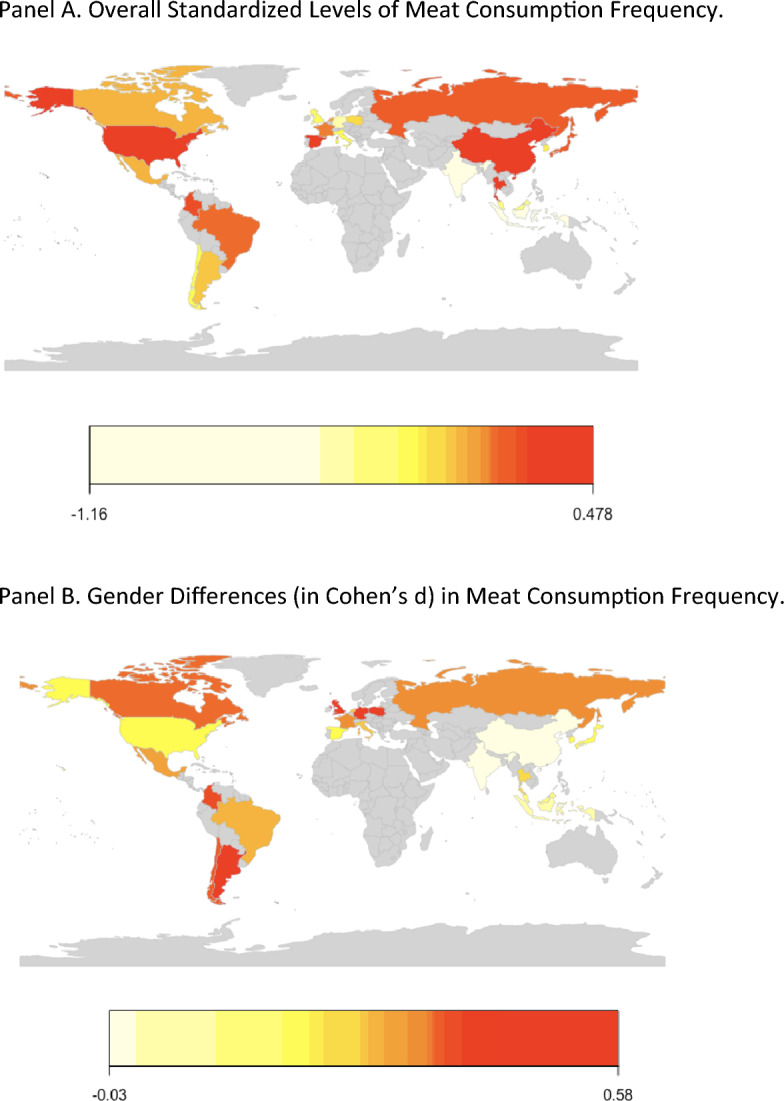
Overall levels (**A**) and gender differences (**B**) in meat consumption frequency across sampled countries. For gender differences, redder color indicates higher levels of consumption frequency for men. (**A**) Overall Standardized Levels of Meat Consumption Frequency. (**B**) Gender Differences (in Cohen’s d) in Meat Consumption Frequency.

**Figure 2:**
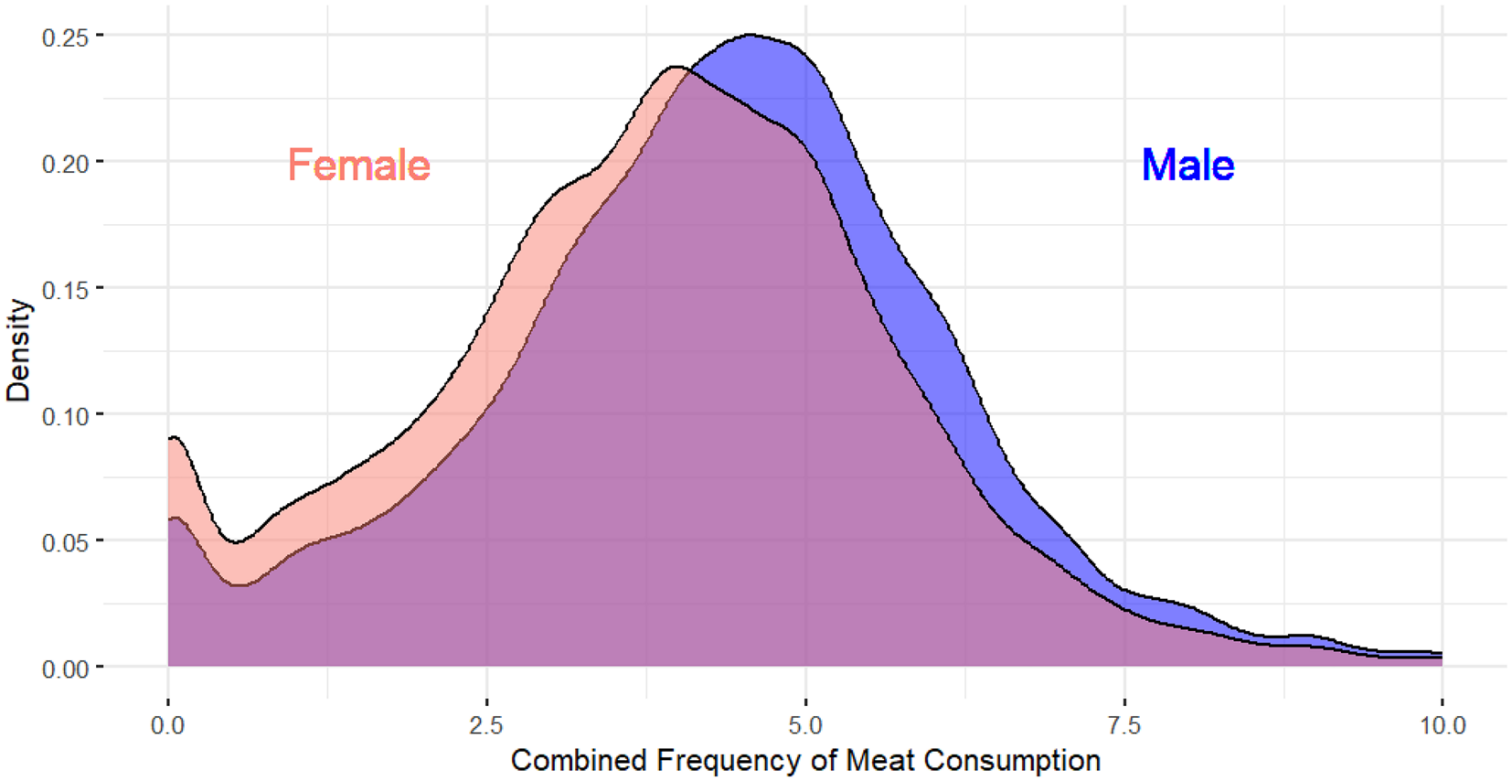
Overall differences in meat consumption frequency across genders.

Table 1 shows the sample sizes, gender proportions, scores on human development and gender equality variables, and meat consumption frequency levels by men and women across countries. With regard to meat consumption, this table shows that, with three exceptions (China, Indonesia, and India), men eat meat more often than women. Significant positive effect sizes ranged from *d* = 0.17 (Malaysia) to 0.58 (Germany). Overall levels and gender differences in meat consumption frequency across countries are depicted in Fig. 1, and overall differences in meat consumption frequency across gender are depicted in Fig. 2.

The intercept-only model explained 11% of the total variance in meat consumption frequency. The random-intercept model explained significantly more variance than the intercept-only model (χ^2^ = 561, *p* < 0.001), justifying the inclusion of gender, age, and age squared as predictors. Age had significant linear (*r* = − 0.07, *t* = − 9.95, *p* < 0.001) and quadratic (*b* = − 0.02, *t* = − 3.77, *p* < 0.001) effects in our preliminary analyses and final models (*p* < 0.001) and thus was retained throughout our model building process. In the model with random coefficients for the slopes of gender, age, and the quadratic age term, including random age effects resulted in model convergence issues. We therefore concluded that the slopes of the age effects did not differ across countries. However, the model that included only the gender slope explained significantly more variance than the random intercept model (χ^2^ = 53.1, *p* < 0.001).

Models that included level 2 variables for gender equality and human development as well as cross-level interactions between gender with human development and gender equality explained significantly more variance than the simpler nested random coefficients model (χ^2^ = 10.6, *p* < 0.005 and χ^2^ = 15.3, *p* < 0.001, respectively). Coefficients for these models can be found in Table 2. Main effects were as expected. Parameter estimates indicated that men consume meat significantly more often than women, meat consumption generally decreases with age but is highest in young to middle-aged adults (the highest average level across all countries was for age 32), and that human development and gender equality are both positively related to meat consumption.

**Table 2:** Predictors of meat consumption frequency in final models.

Fixed parameters	Country-level moderator					
	Human development			Gender equality		
	*r*	95% CI	*p*	*r*	95% CI	*p*
γ_00_ (intercept)	0.17*	[0.03, 0.30]	0.015	0.18*	[0.04, 0.31]	0.011
γ_10_ (gender_*ij*_)	− 0.28***	[− 0.33, − 0.23]	< 0.001	− 0.28***	[− 0.33, − 0.24]	< 0.001
γ_20_ (age_ij_)	− 0.07***	[− 0.09, − 0.06]	< 0.001	− 0.07***	[− 0.09, − 0.06]	< 0.001
γ_30_ (age_ij_^2^)	− 0.02***	[− 0.03, − 0.01]	< 0.001	− 0.02***	[− 0.03, − 0.01]	< 0.001
γ_01_ (moderator_j_)	0.18**	[0.04, 0.31]	0.009	0.17*	[0.03, 0.30]	0.014
γ_11_ (moderator_j_) × (gender_ij_)	− 0.07**	[− 0.12, − 0.02]	0.003	− 0.09***	[− 0.13, − 0.05]	< 0.001

Random parameters	σ^2^	σ^2^				
μ_0j_ (countries)	0.104	0.107				
μ_1j_ (gender_ij_)	0.010	0.007				
r_ij_ (residual)	0.866	0.931				
Pseudo-R^2^ moderator × gender	R^2^ = 32.47%	R^2^ = 52.60%				

Gender effects for female gender. Dependent variable was the Combined Land Animal Consumption Score. HDI (human development) and GGGI (gender equality) were centered on the global mean. Age was grand-mean centered. Explanation of parameters: see Eqs. (1)–(4). The range of HDI and GGGI does not exceed 0.4 in the sample. Pseudo-R^2^ represents the increase in explained residual variance of gender_ij_ through the inclusion of the respective moderator (35). *p < 0.05 **p < 0.01 ***p < 0.001.

**Figure 3:**
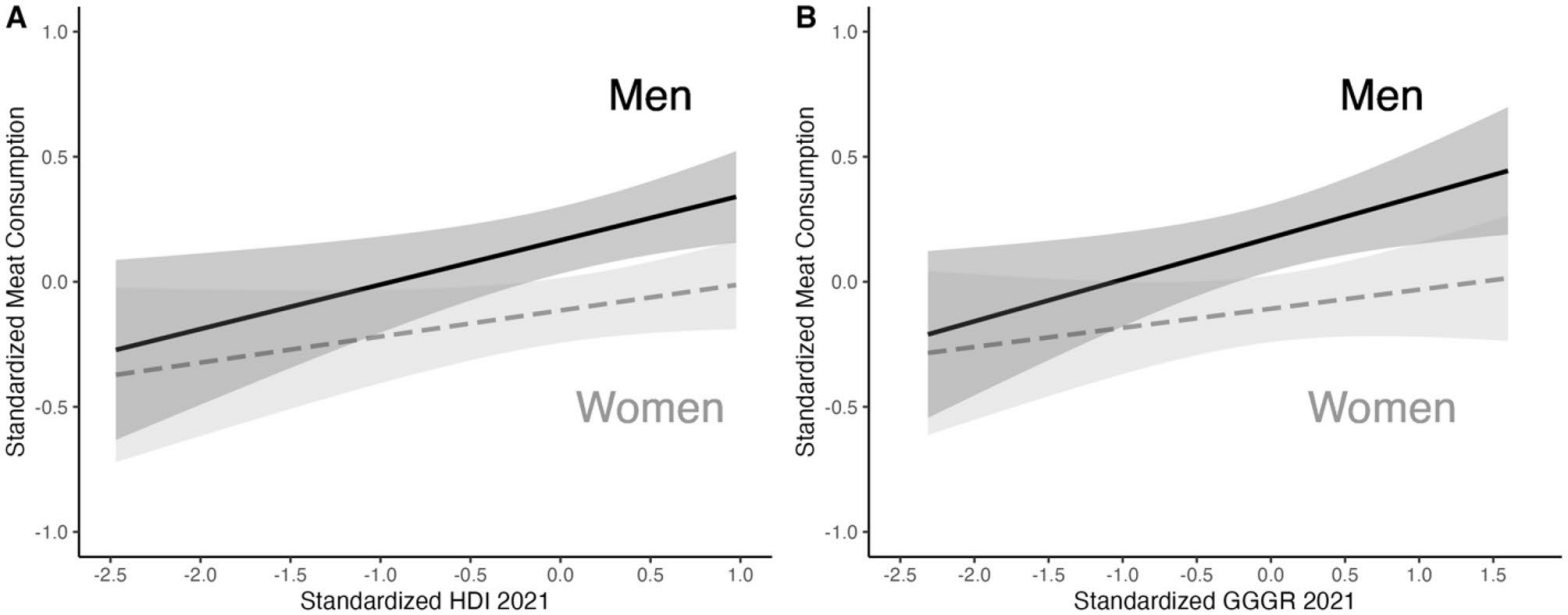
Gender differences in meat consumption frequency are greater in countries with higher human development (**A**) and gender equality (**B**). Country-level predictors centered at the global mean.

Cross-level interactions indicated that gender differences in meat consumption are larger in countries with greater human development and gender equality, consistent with the gender paradox hypothesis. These interactions are depicted in Fig. 3. In supplemental analyses with separate models for each gender group, men’s meat consumption frequency increases significantly with higher gender equality and human development, whereas women’s does not (this is also apparent in Fig. 3).

## Discussion

In this study, we used data from 23 countries from four continents to test gender differences in meat consumption frequency across cultures. As expected, we found that men tend to eat meat more often than women, and that people in more developed countries eat meat more often than people in less developed countries. Of primary interest was the interaction between gender and culture. Similar to previous research with attributes such as personality and self-esteem, we found greater gender differences in meat consumption in countries with higher levels of gender equality and human development. This finding supports the paradoxical gender effect and extends it to a variable that is less prone to reference group effects.

A large body of research now supports that both male gender and wealth are related to greater meat consumption^9,27^. With regard to gender, these results cannot parse various possible explanations for this gender difference, which may range from those involving biological processes to social norms and stereotypes. However, we note that gender differences were not observed in three of the world’s largest countries, India, Indonesia, and China. This suggests that gender differences are not universal and that cultural and contextual factors may play a role. The human development effect is relatively easy to explain by economic factors. Producing meat is more expensive than producing plant-based food for a variety of reasons^35^. It follows that countries in which people have more economic resources afford greater opportunities for people to purchase and consume meat.

Perhaps the most interesting finding from this study is that gender differences in meat consumption frequency are greatest in countries with the highest level of human development and gender equality. It is most intuitive to expect that, as economies develop and particularly as gender roles equalize, gender differences in attitudes and behavior should decrease. However, an emerging body of research across psychological attributes paradoxically suggests that gender differences actually increase in countries that afford women relatively greater opportunity in terms of both wealth and gender norms^21–24^. The extension of this effect to a more behavioral outcome that is rated in terms of frequency rather than relative anchors (e.g., false to true) helps rule out reference group effects as an explanation for this finding.

The finding that this effect is largely driven by increasing meat consumption among men is of considerable interest. Schmitt et al.^22^ suggested that this kind of effect can be attributed to the emergence of natural, dispositional differences in behaviors, attitudes, and preferences in cultures that afford women greater freedom and choice. For instance, in countries relatively high in both human development and gender equality, there may be more options for satisfying non-meat foods and lower expectations that women will share the same preferences and food choices as men. However, our data suggest that the effects of human development and gender equality on gender differences in meat consumption have more to do with men’s consumption behavior than women's, meaning that this effect is more likely to be driven by more extensive meat consumption among men in developed countries, in which greater wealth creates more opportunities for men to choose meat, than by lower meat consumption among women. To the degree that various factors lead men to prefer meat more than women, these preferences could thus be both universal and expressed more easily in contexts with greater opportunity for independent choices by men.

Meat consumption is a significant contributor to climate change^36^, pandemic risk^37^, clean water shortages^38^, and social injustice for farmed animals^3^. Therefore, it is in society’s interest to curb meat consumption worldwide. These findings may hold some clues as to the most effective means of reducing meat consumption. For instance, interventions targeting individual behavior may be tailored based on gender and gender identity, and to focus on meat reduction among men^31^. Meat reduction strategies at a society level may benefit from considering context^39–41^. Countries with highest levels of consumption and highest human development offer consumers the most choice, including opportunities to consume plant-based or cultivated alternatives to farmed animals. In such countries, demand-side approaches involving marketing or other consumer-targeted strategies may be most effective. In contrast, meat consumption is increasing the most in developing countries, but these countries are also likely to be hardest hit by animal agriculture because it is economically inefficient and because such countries tend to be more vulnerable to environmental and social justice challenges. Thus, supply-side strategies involving incentives to produce plant-based alternatives and cultivated meat may be most effective.

## Limitations

This study was affected by several limitations. First, we assessed a limited range of countries. Sampling issues may have influenced results and limited the sensitivity of our models to country-level effects, such as differences between the impacts of gender equality and human development. Moreover, no African countries were represented at all, and it is possible that gender and culture have qualitatively different impacts on meat consumption in an African context. Second, we counted the number of meals in which meat was eaten, but not self-reported caloric intake. Studies with more direct and objective measures of meat consumption should be conducted to replicate these results. Third, we classified people according to self-reported gender, and cannot draw conclusions about people who do not identify as men or women. Fourth, we did not examine different types of meat. Given how cultural and economic factors influence meat consumption, this is an important nuance to consider in future studies.

## Conclusion

In addition to replicating findings that men tend to eat more meat women and that more meat is consumed in more developed countries, this study is the first to document paradoxical gender effects in meat consumption. Specifically, gender differences in meat consumption are greater in countries with higher human development and more gender equality. This finding builds upon similar studies with psychological attributes, and helps rule out reference group effects as an explanation. This study also provides information that may be useful in thinking about how to reduce meat consumption worldwide.

## Data Availability

Data and materials for this study are available at https://osf.io/z37w6/?view_only=a469a98d605046df890bfcf5f0f5d6c7. Codebook for the overall study is available at https://osf.io/tx3u9/?view_only=07504017687249d3bb05d93db9c83914.
